# Next-generation sequencing in NSCLC and melanoma patients: a cost and budget impact analysis

**DOI:** 10.3332/ecancer.2016.684

**Published:** 2016-10-28

**Authors:** Rosa A van Amerongen, Valesca P Retèl, Veerle MH Coupé, Petra M Nederlof, Maartje J Vogel, Wim H van Harten

**Affiliations:** 1 Department of Epidemiology and Biostatistics, VU University Medical Center, PO Box 7057, 1007 MB Amsterdam, The Netherlands; 2 Department of Psychosocial Research and Epidemiology, The Netherlands Cancer Institute, PO Box 90203, 1006 BE Amsterdam, The Netherlands; 3 School of Governance and Management, University of Twente, MB-HTSR, PO Box 217, 7500 AE Enschede, The Netherlands; 4 Department of Molecular Diagnostics, Pathology, The Netherlands Cancer Institute, PO Box 90203, 1006 BE Amsterdam, The Netherlands

**Keywords:** Next-generation sequencing, test costs, budget impact, melanoma, NSCLC, personalised medicine, targeted therapy

## Abstract

Next-generation sequencing (NGS) has reached the molecular diagnostic laboratories. Although the NGS technology aims to improve the effectiveness of therapies by selecting the most promising therapy, concerns are that NGS testing is expensive and that the ‘benefits’ are not yet in relation to these costs. In this study, we give an estimation of the costs and an institutional and national budget impact of various types of NGS tests in non-small-cell lung cancer (NSCLC) and melanoma patients within The Netherlands. First, an activity-based costing (ABC) analysis has been conducted on the costs of two examples of NGS panels (small- and medium-targeted gene panel (TGP)) based on data of The Netherlands Cancer Institute (NKI). Second, we performed a budget impact analysis (BIA) to estimate the current (2015) and future (2020) budget impact of NGS on molecular diagnostics for NSCLC and melanoma patients in The Netherlands. Literature, expert opinions, and a data set of patients within the NKI (*n* = 172) have been included in the BIA. Based on our analysis, we expect that the NGS test cost concerns will be limited. In the current situation, NGS can indeed result in higher diagnostic test costs, which is mainly related to required additional tests besides the small TGP. However, in the future, we expect that the use of whole-genome sequencing (WGS) will increase, for which it is expected that additional tests can be (partly) avoided. Although the current clinical benefits are expected to be limited, the research potentials of NGS are already an important advantage.

## Background

Next-generation sequencing (NGS) has reached the molecular diagnostic laboratories and is expected to (slowly) replace single-gene molecular diagnostic tests for the detection of DNA mutations in tumour tissues [1]. Within The Netherlands, currently, many (specialised) hospitals and university medical centres have implemented NGS for diagnostics in the clinic. NGS can be used to identify multiple specific mutations in a tumour, to which genomic-directed targeted therapy (hereinafter referred to as targeted therapy) may be assigned, as standard-of-care or referral to a clinical trial. A NGS panel can be described as ‘a multiplex predictive test, which explores limited regions of tumour DNA/RNA for aberrations that can be used as a molecular target for therapy’ [2]. There are several types, such as single-gene tests, small- (~50 genes, using most often a polymerase chain reaction (PCR)-based technique), medium- (~200 genes, using DNA captures), and large (using e.g., whole-exome sequencing (WES))-targeted gene panels (TGP), and whole-genome sequencing (WGS) [3]. The tests vary in price and turnaround time, and the design of the panels determines the different targeted therapy options.

There are high expectations around NGS and targeted therapies, they can improve patient survival and can reduce unnecessary harms and costs by preventing sequential single-gene testing and ineffective treatments [4–5]. For research, NGS is expected to speed up patient selection in clinical trials and to increase knowledge concerning the prognostic impact of targets [6–7]. In The Netherlands, an initiative was started by the Hartwig Medical Foundation (HMF) within the ‘Centre for Personalised Cancer Treatment (CPCT)’. The CPCT has the mission to serve all Dutch cancer patients with testing WGS for diagnostics. The CPCT is a nationally operating consortium involving all nine academic medical centres in The Netherlands (all UMC’s and The Netherlands Cancer Institute (NKI)) and an increasing number of teaching hospitals (STZ). The ultimate goal is to serve both direct patient diagnostics towards experimental care (clinical trials, off-label drug use) and the construction of a nationwide database with sequencing and treatment response information.

Currently, molecular diagnostics and targeted therapies are mostly recommended for patients in an advanced stage of disease. Especially, for stage-IV non-small-cell lung cancer (NSCLC) and stage-IV melanoma patients, NGS can be very beneficial due to their heterogeneous nature, high mutation frequency, and the availability of targeted therapies. For both patient groups, the prognosis is poor, stage-IV NSCLC patients have a 5-year survival rate of 1% and stage-IV melanoma patients of 15–20% [8–9]. Since 2004 for NSCLC and 2011 for melanoma, several targeted therapies are approved by the European Medicines Agency (EMA) [10]. These therapies show promising results, overall response rates above 60% are measured in both NSCLC and melanoma patients treated with targeted therapy, compared with 20% by chemotherapy [11–12]. In case of no actionable mutation, which is currently the case in approximately 78% of the NSCLC and 49% of the melanoma patients, usual chemotherapy or immunotherapy is recommended [13]. In the current Dutch diagnostic guidelines, still only the testing of two single genes are recommended for stage-IV NSCLC patients (epidermal growth factor receptor (EGFR) and the anaplastic lymphoma kinase (ALK)) [14]. For stage-IV melanoma patients, so far, no molecular diagnostic test is recommended, although a single-gene test investigating the BRAF V600E-gene (BRAF) mutation status is often applied [15]. However, as seen in the NKI, specialised hospitals already use more extensive molecular diagnostic tests for both patient groups.

Although the NGS technology aims to improve the effectiveness of therapies, concerns are that NGS testing is expensive and that the clinical benefits are not yet in relation to these costs [16]. Currently, there are publications on cost-effectiveness analyses of NGS available [17–18]. However, these publications do not contain detailed cost analyses regarding the sequencing tests themselves. In order to support the decision whether the implementation of NGS should be prolonged or extended within the clinic, our department started an early health technology assessment (HTA), with a scenario analysis focused on NGS implementation by means of expert elicitation [2]. Subsequently, explorations of the potential costs and benefits are needed, starting with the costs of NGS and expected developments. The objective of the current study was firstly, to calculate the costs for the small- and medium TGP used within our institute (NKI) and secondly, to estimate the budget impact of the old, current and future situation around NGS and other molecular diagnostic test costs from a national perspective (Dutch), and an institutional perspective (NKI).

## Methods

### Activity-based costing: test costs

The total costs of performing two types of NGS tests for diagnostics have been analysed for a small TGP (48 genes) and a medium TGP (178 genes). For this, we used activity-based costing (ABC), a cost calculation technique that links resource costs with products using a multistep allocation procedure on the basis of activity consumption [19]. This practically means that for each product, in this case a diagnostic test, all required activities that consume resources, varying from consumables to personnel time, are included in the calculation. The cost calculations for the two types of TGPs were based on NKI data. A small TGP, targeting 48 genes and 212 amplicons on DNA level (the TruSeq Amplicon Cancer Panel (TSACP) from Illumina BV), was recently implemented in routine diagnostics. The medium TGP was an in-house developed capture panel covering the full exons of 178 cancer-related genes, using a customised SureSelect XT2 capture library and magnetic streptavidin beads for capture. This panel can be used for the detection of both DNA and RNA variants, but in this study, only the DNA part is considered. Both the procedures can be executed on the Illumina Miseq^TM^ and the Illumina Hiseq^TM^, and per run a maximum of 48 samples can be included, except for a run with the medium TGP on the Miseq^TM^ which is restricted to four samples per run. These maximums of four or 48 samples per run are related to the desired minimum depth of coverage and pipetting time needed per sample.

The test costs consisted of the following elements: personnel, material, equipment, and an estimation of the overhead costs. Personnel costs were based on the estimated minutes spent per process step, both per sample and run. Hour rates per personnel type were based on the NKI employers’ costs of 2014 and only the direct productive hours were included in this calculation. Real ‘timing activity’ was used to calculate the time required for the small TGP currently in clinical practice. Since the medium TGP was not used in a diagnostic setting, timing estimations were made by means of interviews with the executers of the process. Material costs were based on laboratory protocols and purchase prices within the NKI. Laboratory protocols were also used to calculate the equipment costs; realistic redemption durations and yearly service costs were taken into account. All costs were divided into fixed yearly costs, fixed costs per run and variable costs per sample. The cost consequences of important parameters of the NGS procedure were explicitly investigated; the type of sequence technology (Illumina Miseq^TM^/Hiseq^TM^), the numbers of samples per run, the number of runs per week and the completeness of the run. Variation in these parameters will result in different molecular diagnostic test costs and turnaround times of the diagnostic process.

### Budget impact analysis

A budget impact analysis (BIA) was performed to estimate the yearly budget impact of NGS tests in NSCLC and melanoma patients from a Dutch perspective and an institutional perspective (NKI). A BIA focuses on the expected changes in the expenditure of a health care system after the adoption of a new intervention over a time period of 5 years in this case. Time frames 2012, 2015, and 2020 have been selected to show an estimation of the old, current and future budget impact. In 2012, mostly (a combination of) single tests and methods were used in clinical diagnostics. In 2015, a variety of NGS tests were represented from single-gene tests in the general hospitals, till small-medium TGPs in the academic centres. In the future time frame of 2020, it was assumed that WGS will be the clinical standard in academic/specialised hospitals by that time. The latter assumption is in the framework of the CPCT, their mission is to perform WGS in all specialised centres, for both diagnostics and research.

**Figure 1.**
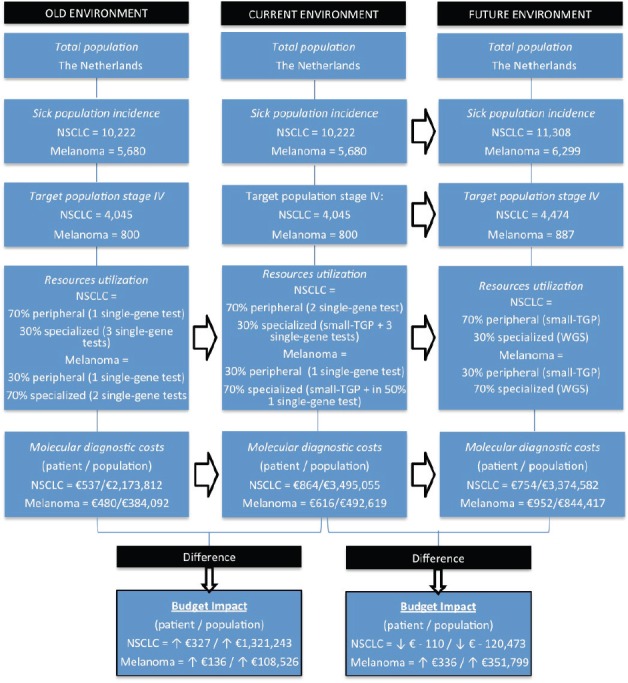
Budget impact analysis model, showing the aspects within the ISPOR framework for the old, current, and future environment. The arrows indicate a change of one of the aspects overtime. The target populations are divided into patients receiving peripheral and specialized care, based on the type of hospital they are diagnosed. At the bottom, the average cost differences per patient and population between the old and current environment and current and future environment are shown for both NSCLC and melanoma patients. NSCLC = non-small-cell lung cancer, NGS = next-generation sequencing, WGS = whole-genome sequencing.

Within our BIA, six sources of input are included: three test cost sources, literature for diagnostic guidelines and patient numbers, patient data for the case study and expert opinions. The BIA has been performed according to the BIA framework of the International Society for Pharmacoeconomics and Outcomes Research (ISPOR) [20]. This framework consists of several standard aspects: target population, scenario distribution based on hospital types, resource utilisation, costs per unit, total costs, and sensitivity analyses. Our BIA model is shown in Figure 1 and the different aspects are discussed below.

#### Target population and scenario distribution (nationwide)

The NSCLC and melanoma incidence within The Netherlands and the stage distribution at diagnosis were used to calculate the size of the target population, stage IV NSCLC and melanoma patients within The Netherlands. The patients were divided into two groups: peripheral or specialised healthcare (the latter including NKI, university medical centres and other specialised hospitals), according to the number of patients diagnosed per hospital type. Different molecular diagnostic test standards have been taken into account for these two types of hospitals. We assumed that for each new stage-IV patient, molecular diagnostics is executed once. Although repeated testing over time is often required in case of progression, among others caused by tumour heterogeneity within a patient, we assumed no influence by NGS on this frequency.

#### Resource utilisation

The resource utilisation within this BIA reflects the use of molecular diagnostic tests (including personnel, material, and equipment use) within the Dutch health care system. Based on the Dutch diagnostic guidelines [14–15], the clinical standard of molecular diagnostics in peripheral hospitals has been estimated. The two used diagnostic guidelines for NSCLC patients for the old and current situation were implemented in, respectively, 2011 and 2015. For melanoma patients, no molecular diagnostic test is included in the guideline yet; however, since 2012 a single-gene test is recommended, and therefore, we assume this test to be the clinical standard in peripheral hospitals. We made the assumption that in all specialised hospitals the old and current clinical standard of molecular diagnostics was similar as within the NKI. The test scenarios in the future situation, including expected patient numbers for which NGS is requested, process improvements and renewed gene panels, are based on expectations within the NKI. We assume in our analysis that no other changes in the general executed diagnostics and imaging will occur. We also assume biopsy- and test failure rates to stay similar in the future.

#### Costs per unit

Three different sources have been used to estimate the molecular diagnostic test costs. (1) The costs for single-gene tests were estimated within the NKI and it was assumed that these costs are comparable in other hospitals. (2) The small- and medium-TGP costs were based on the ABC analysis explained in the “Activity-based costing: test costs” section. (3) The WGS costs estimation was based on the public data of the National Human Genome Research Institute, showing the most recent sequence costs per DNA sequence or genome [21], and the future estimation was discussed with experts in The Netherlands. We assumed that the current costs of molecular diagnostic tests in clinical practice are representative for future costs as well. However, for the WGS costs, currently still under development with major cost reductions over the past years, we assume a reduction of 75% in five years.

#### Sensitivity analysis

Considering the fact that many included parameters for the BIA were based on hypothetical values and extrapolations, plausible ranges for the model’s parameters were explored. We conducted two one-way sensitivity analyses, one for the test costs in the current NGS situation of NSCLC and one for the influence of future test costs on the budget impact. We varied key parameters one-by-one, using several uncertainty ranges. For the test costs, four parameters have been included: (1) Number of NGS samples per week, (2) Number of NGS runs per week, (3) Completeness of the NGS run, and (4) Required additional tests besides NGS. All base values and ranges were based on realistic possibilities within the NKI. Three treatment parameters were included in the sensitivity analysis for the future budget impact: (1) Price of TGP using NGS, (2) Price of WGS, and (3) Percentage of patients that will receive specialised care (using WGS testing). For these parameters, a range of 20% has been used, as seen in other comparable BIA’s [22].

#### Institutional perspective (case study): BIA within the NKI

In order to analyse the budget impact of NGS in clinical practice, the NKI was used as a case study environment. Stage-IV NSCLC and melanoma patients for who molecular diagnostics were requested in a period before NGS implementation (November–December 2014) and in a period after NGS implementation (January–February 2015) were included. A data set was created with the diagnosis, number, and type of molecular diagnostic tests per patient and the observed mutations per test. Both actionable and non-actionable mutations were counted and the percentages of patients with an actionable mutation for which hypothetically a targeted therapy is available (EMA approved or in trial) were calculated. Treatment consequences were manually checked via the electronic health records of the patients and four categories were added to the data set: (1) whether there was a treatment option for the patient, (2) which treatment was selected, (3) whether the selected treatment was based on the test result, and (4) whether the patient received a first dose of the treatment. The molecular diagnostic tests, test results, and treatment consequences have been compared for the two periods. Based on the molecular tests executed per patient, the test costs per patient and for the total NKI population were calculated, including a budget impact of the NGS implementation. We assumed that the number of included patients in these 3.5 months was representative for the rest of the year, in order to estimate the yearly NKI population of NSCLC and melanoma patients for who molecular diagnostic tests are requested.

#### Analysis

Microsoft Excel and SPSS 23.0 have been used to perform the sensitivity analyses and to create descriptive statistics of the results. An independent two-sample *t*-test for the continuous variables with a normal distribution, an independent samples Mann–Whitney U-test for the continuous variables without a normal distribution and a Fisher’s exact test for the binary variables have been used to analyse differences between the subgroups of the NKI case study. *P* values below 0.05 were considered to indicate a statistically significant difference.

## Results

### Activity-based costing: NGS costs

**Figure 2.**
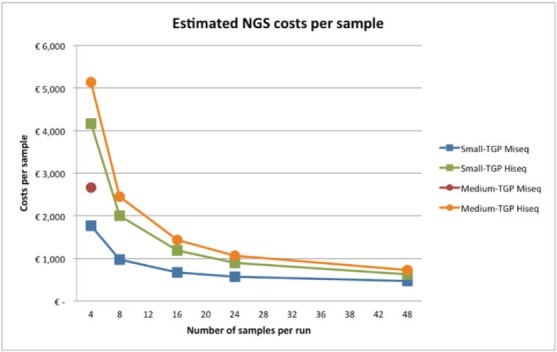
Estimated costs per sample, dependent on the number of samples per run, assuming one run per week, and complete runs. The costs are shown for both the small- and medium TGP on the Illumina Miseq^TM^ or Hiseq^TM^. There is a maximum of four samples for the medium TGP on the Illumina Miseq^TM^, therefore only one data point is given. NGS = next-generation sequencing, TGP = targeted gene panel.

Figure 2 shows the estimated costs per sample dependent on the number of samples per run. A higher number of samples per run resulted in lower costs per sample, due to the fixed costs per year and fixed costs per run. The following mean costs per sample are given for a run with 24 samples, one run executed per week and on average 85% of the run filled. Between brackets the minimum costs with 48 samples and maximum costs with four samples are shown, respectively. Costs per sample with the small TGP were €606 (€465; €1769) on the Miseq^TM^ and €956 (€621; €4,161) on the Hiseq^TM^. A run with the medium TGP on the Miseq^TM^ is restricted to four samples per run, for €3009 per sample, and costs per sample on the Hiseq^TM^ were €1,137 (€857; €5273). These costs are inclusive VAT, overhead (30%) and the costs of control samples, in a run with ≤8 samples one control sample is included and in a run with ≥9 samples two control samples are included. A general overview of the costs is shown in Table 1 and Table S1 and S2 give a more comprehensive overview of the included costs.

### Budget impact analysis

#### Target population and scenario distribution (nationwide)

Within The Netherlands, the current incidence of stage-IV NSCLC and melanoma patients are, respectively, 4,045 and 800 patients per year [23–25]. In the future situation of our BIA, 4,474 NSCLC and 887 melanoma patients are included, based on the expected increase of 11% in 5 years [26]. We assumed that 70% of the NSCLC patients receive their care in a general hospital, the other 30% of patients receive specialised care [24]. Since 2013 it has been determined that all stage-IV melanoma patients have to be referred to specialised melanoma centres (14 specialised hospitals in The Netherlands). This results in a distribution where 70% of the patients are treated in a specialised hospital and 30% in a peripheral hospital [27].

#### Resource utilisation

An overview of the specific test types per time frame and hospital type, including the costs per unit as discussed in the next part, is given in Table 2. In the old situation, in peripheral hospitals, we assumed one single-gene test as clinical standard for both NSCLC (EGFR) and melanoma patients (BRAF). In the current situation for NSCLC, we assumed one additional single-gene test (ALK), based on the current guidelines [14–15]. In the old situation within specialised hospitals, we assumed that multiple single-gene tests were performed, minimally three for NSCLC patients (a multigene panel, HER2/EGFR, and ALK, ROS, RET, or MET) and two for melanoma patients (BRAF and NRAS or KIT). In the current situation in specialised hospitals, small–medium TGPs are increasingly implemented. Based on our experience in the NKI, it is estimated that, especially for NSCLC, additional tests will be required due to (temporary) inefficiencies and due to specific translocations not included in the small TGP. Therefore, besides the small TGP, three additional single-gene tests were assumed for NSCLC patients (e.g., fragment analysis (HER2/EGFR) to detect larger deletions/insertions, Sanger sequencing (EGFR) and fluorescence *in situ* hybridisation (ALK, ROS, RET, or MET)) and one single-gene test was assumed for 50% of the melanoma patients (Sanger Sequencing (KIT)). In the future (2020), we assumed in our model that NGS with a small TGP will be clinical practice in peripheral hospitals for both NSCLC and melanoma patients. In specialised hospitals, we assumed WGS to be clinical practice, following the developments of the CPCT. For both NGS and WGS, we assumed in the future no required additional tests caused by inefficiencies.

#### Costs per unit

In peripheral hospitals, the average diagnostic test costs per NSCLC patient in the old, current, and future situation were, respectively, €295; €590, and €606. In the specialised hospitals, the test costs were, respectively, €1103; €1504, and €1100. For melanoma patients in peripheral hospitals, these costs were, respectively, €295, €295, and €606 and in specialised hospitals €559, €753, and €1100. In Table S3, an overview of the costs per test is shown, including the cost source and an explanation for the WGS costs estimation.

#### Total costs

The yearly budget impact for NSCLC patients was as follows: the current situation will result in a test cost increase of €327 per patient, followed by a cost decrease of €110 in the future situation. For the total population in The Netherlands, this will be, respectively, an annual increase in €1,321,243 and annual decrease in €120,473. For melanoma patients, an increase of €136 and €336, respectively, in test costs per patient is expected, resulting in a yearly budget impact of €108,526 and €351,799 for the total population. A summary of the BIA results is given in Table 3.

#### Sensitivity analyses

**Figure 3.**
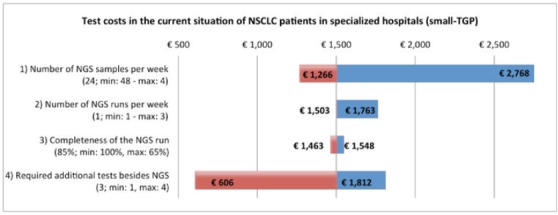
One-way sensitivity analysis on the effect of four parameters on the test costs in the current situation of NSCLC patients in specialised hospitals. TGP = targeted gene panel.

**Figure 4a and b.**
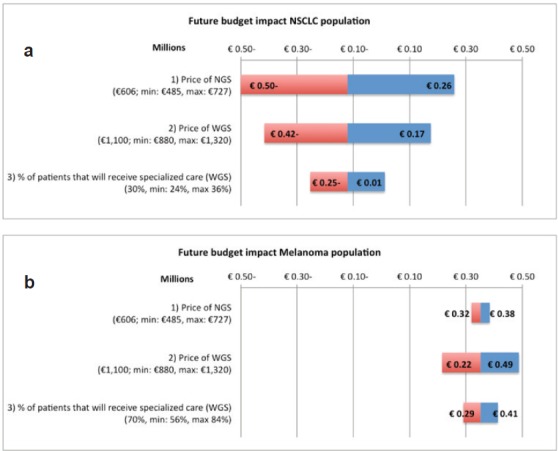
One-way sensitivity analysis on the effect of three parameters on the budget impact of the future scenario. The amounts on the x-as are shown in millions and account for the Dutch NSCLC patient population (4a) and the Dutch melanoma patient population (4b). NSCLC = non-small-cell lung cancer, NGS = next-generation sequencing, WGS = whole-genome sequencing.

The sensitivity analysis of the test costs in the current NGS situation of NSLCC in specialised hospitals showed a range of €606 to €2614. For the base value, €1504 as shown in the ‘costs per unit’ section, it is assumed that per NGS run 24 samples are included, per week one run is executed, on average 85% of the run is filled and three additional single gene tests are required. The biggest reduction in test costs can be achieved by a reduction in additional tests besides NGS. The base value, range, and the effect on the test costs are shown for each parameter in the tornado plot in Figure 3. The sensitivity analysis of the budget impact of the future scenario compared with the current situation shows for the NSCLC population a range of minus €500,080 to plus €259,134. For the melanoma population a range between €215,173 and €488,424 has been calculated. The base values, minus €120,473 (NSCLC) and €351,799 (melanoma), are based on the results of our BIA calculation, discussed in the ‘total costs’ section. The price of NGS shows the widest range in the budget impact for the NSCLC population. For the melanoma population, the biggest reduction can be achieved by a reduction in the price of WGS. The tornado plots of these analyses are shown in Figure 4.

#### Institutional perspective (case study): BIA within the NKI

In total 172 patients were included, all stage-IV NSCLC and melanoma patients within the chosen time periods have been selected. The test-related measures, mutation, and treatment characteristics for the period before and after NGS implementation are shown in Table 4. In NSCLC patients, there is only a significant increase in the number of tests and tests costs after the implementation of NGS. In melanoma patients, significant differences have been observed both in test-related measures and mutation characteristics: the number of tests per patient dropped after the NGS implementation and the number of observed mutations increased. The test costs per patient and for the total patient population in the period before and after NGS implementation are shown in Table 5. Yearly 343 stage-IV NSCLC patients and 247 stage-IV melanoma patients within the NKI were assumed. The NGS implementation resulted within the NKI in a budget impact of an additional spending of €357 per NSCLC patient and a saving of €14 per melanoma patient. For the total NKI population, this meant a budget impact of €119,087 (€122,498 increase for NSCLC patients and €3,411 savings for melanoma patients).

## Discussion

As far as we know, this is one of the first published cost analysis and budget impact analysis regarding NGS. As expected, our results showed that the more samples are included and runs are performed, the less costly NGS will become. Regarding budget impact, we showed that currently NGS resulted in an increase in test costs, from 2012 to 2015 yearly €1.32 million for the total NSCLC population and €0.11 million for the total melanoma population. The finding of these current budget impacts was confirmed by the NKI case study. From the current to future situation, our BIA showed for the total NSCLC population an expected annual decrease of €0.12 and for the total melanoma population an increase of €0.35 million. This decrease in NSCLC patients was related to the more efficient use of testing by WGS in specialised hospitals, without the need of additional tests. The increase in future test costs for melanoma patients was related to both the implementation of NGS in peripheral hospitals and the implementation of WGS in specialised hospitals, while no additional test costs were involved. Our sensitivity analyses produced widely varying budget impact estimates in the future; especially the price of the small TGP in NSCLC and the price of WGS in melanoma patients had a high impact.

The ABC analysis produced a wide range for the estimate of NGS test costs depending on the number of samples per run, number of runs per week, device, and type of TGP. These calculated test costs are comparable with other recently published cost analysis of NGS techniques. For example, in a situation with six samples per run 600–900 USD was calculated for a similar small TGP and 2000 USD for a medium TGP [28]. Policy decisions in institutions have to be made which panel type and number of runs per week will be most beneficial for patients, regarding both the costs and consequences. For example, the decision whether an increase in the number of mutations tested can outweigh the higher costs and turnaround time. In a macro level, although the current costs in combination with the benefits of NGS are still unclear, NGS especially has a lot of potential for the future. Firstly, NGS is expected to replace single-gene tests, which can reduce the costs and limits patient material use [5]. Currently, additional tests are still needed to supplement the small TGP test; however, it is expected that eventually these additional tests become (partly) superfluous. The research potentials of NGS are an important potential advantage as well [6–7]. A broader panel of genes can result in more treatment options for patients, both off-label and in clinical trial. This can speed up patient recruitment for clinical trials and consequently targeted therapies can become EMA approved more rapidly. These potentials are relevant both for current and future patients.

Our analyses have some limitations. Firstly, we made several assumptions in the BIA. In the future situation, we assumed no inefficiencies of NGS or WGS that require additional tests and no findings of new clinical relevant modifications not traceable with WGS. We also assumed that the current costs of molecular diagnostic tests in clinical practice are representative for the future and we have made an estimation for the WGS costs reduction in five years. The second limitation of our BIA is the fact that the consequences of NGS are not taken into account; in particular, treatments costs can have a major impact on the total budget. By identifying more targetable targets, NGS can result in an increased use of targeted therapies [29]. However, without clinical data, it is very complicated to estimate the treatment consequences. In clinical practice, patients receive several treatment lines and several factors, besides mutation status, play a role in therapy selection or whether a treatment is prescribed at all [30]. Furthermore, it is hard to estimate the availability of new targeted therapies, their prices and average treatment durations in the future. The third limitation of our study is related to the generalisability of our NKI case study and the clinical practice within the NKI, which we used to estimate the test situation in specialisd hospitals within The Netherlands. The NKI is a specialised comprehensive cancer centre, with a broader variety of molecular diagnostic tests and a higher number of clinical trials. The patient population is different within the NKI as well; patients are often redirected to the NKI after an ineffective first-line treatment elsewhere. In addition, a larger data set would have been desirable, however, this was not feasible due to the recent implementation.

Nevertheless, the NKI case study showed interesting and comparable results with our main Dutch BIA. Between the period before and after NGS implementation, only differences in average number of tests and associated costs were found. No significant differences in mutation and treatment characteristics were found, except for the number of observed mutations in melanoma patients. We expected beforehand that no difference in EMA approved targeted therapy use would be observed, since the number of druggable targets is low and within the time period no additional targeted therapies were EMA approved [10]. However, an increase in participation in trials would have been a realistic option, since off label-targeted therapy use is investigated in several trials. Not finding an increase so far can hypothetically be caused by the small data set in combination with the strict inclusion criteria for trials and the recent implementation of NGS, whereby trials are not yet focused on new NGS results. Although no direct clinical benefits of NGS have been measured, the increase in number of observed mutations in melanoma (significant) and NSCLC patients (not significant) is already in line with the expected research potentials of NGS. Furthermore, the test costs in the NKI case study and the main Dutch BIA in specialised hospitals are comparable: per NSCLC patient, respectively, €784 versus €1103 in the old situation and €1141 versus €1504 in the current situation. This applies for the melanoma patients as well, per patient, respectively, €711 versus €559 in the current situation and €698 versus €753 in the future situation. An explanation for the difference in test costs in the current situation is related to the fact that not for all patients in the NKI case study NGS was executed. The selected molecular diagnostic tests are dependent on the request of the clinician or on already known mutation profiles of patients due to previously executed molecular tests.

## Conclusions and implications

Based on our analysis, we expect that the concerns, of NGS resulting in high additional test costs, will be limited. Our BIA does show that NGS currently results in higher costs, which is mainly related to the required additional tests besides the small TGP. However, it is expected that these additional tests become (partly) superfluous in the future. Furthermore, the direct clinical benefits of NGS are currently limited, since no treatment consequences are yet expected due to the low number of druggable targets. Nonetheless, the research potentials of NGS are already an important advantage.

Our cost analysis of NGS and the NKI case study can assist hospitals in the decision which panel or device is most beneficial for them. Based on our analysis, we would suggest a small TGP as currently the best NGS option for diagnostics and we recommend NGS process optimisation to focus on the reduction of additional tests. Our research provides useful input for a health technology assessment of NGS. Interesting follow-up steps would be an extension of our BIA approach to the total cancer population within The Netherlands and the inclusion of clinical data including treatment costs. Furthermore, the inclusion of additional future scenarios with liquid biopsies or a centralised NGS test option within The Netherlands, as proposed by the CPCT/MHF, would be of interest as well.

## List of abbreviations

NGS: Next-generation sequencing
WGS: Whole-genome sequencing
WES: Whole-exome sequencing
NSCLC: Non-small-cell lung cancer
ABC: Activity-based costing
BIA: Budget impact analysis
TGP: Targeted gene panel
NKI: Netherlands Cancer Institute
DNA: Deoxyribonucleid acid
CPCT: Centre for Personalised Cancer Treatment
HMF: Hartwig Medical Foundation
STZ: Dutch teaching hospitals
UMC: University Medical Centre
EMA: European Medicines Agency
EGFR: Epidermal growth factor receptor
ALK: Anaplastic lymphoma kinase
BRAF: Rapid accelerating fibrosarcoma type B oncogene B1
HER2: Human epidermal growth factor receptor 2
KRAS: Kirsten-rat sarcoma 2 viral oncogene homolog
ROS: receptor tyrosine kinase
RET: rearranged during transfection
MET: MNNG-HOS transforming gene
HTA: Health technology assessment
TSACP: TruSeq Amplicon Cancer Panel
ISPOR: International Society for Pharmacoeconomics and Outcomes Research

## Conflicts of Interest

Authors declare no conflict of interest regarding the work described in this manuscript.

## Authors’ contributions

RVA held in-house interviews, collected the input for the cost analysis and BIA, processed the data, analysed the results, and drafted the manuscript. VR held in-house interviews, coordinated the input collection, analysed the results, and co-drafted the manuscript. VC helped to set up the BIA, advised on the results analysis, and critically revised the manuscript. PN provided information on the test activities, process costs, and patient data, gave insight in future expectations and critically revised the manuscript. MJ provided information on the test activities, gave insight in future expectations, and critically revised the manuscript. WVH conceived the work, coordinated the project and helped drafting the manuscript. All authors read, revised, and approved the manuscript.

## Figures and Tables

**Table 1.** A summary of the NGS costs for four options; small or medium TGP on the Illumina MiseqTM or HiseqTM.

	Fixed yearly costs		Fixed costs per run		Variable costs per sample		Total costs per sample	
**Miseq^TM^**	**Small**	**Medium**	**Small**	**Medium**	**Small**	**Medium**	**Small**	**Medium**
Personnel	€ 0.00	€ 0.00	€ 347	€ 840	€ 121	€ 153	€ 606 (€ 465-€ 1,769)	€ 2,668
Material	€ 0.00	€ 0.00	€ 1,198	€ 2,936	€ 155	€ 136		
Equipment	€ 96,589	€ 98,416	€ 0.00	€ 0.00	€ 0.00	€ 0.00		
**Hiseq^TM^**	**Small**	**Medium**	**Small**	**Medium**	**Small**	**Medium**	**Small**	**Medium**
Personnel	€ 0.00	€ 0.00	€ 347	€ 840	€ 121	€ 153	€ 956 (€ 621-€ 4,161)	€ 1,137 (€ 723 -€ 5,133)
Material	€ 0.00	€ 0.00	€ 3,528	€ 5,435	€ 155	€ 136		
Equipment	€ 262,380	€ 264,206	€ 0.00	€ 0.00	€ 0.00	€ 0.00		

The three cost categories are divided into fixed yearly costs, fixed costs per run, and variable costs per sample. The material and equipment costs are inclusive VAT and all costs are exclusive overhead, only the total costs are shown including overhead. In the last column, the total costs per sample are shown for a run with 24 samples and a completeness of the run of 85%. Between brackets the minimum costs for a run with 48 samples and the maximum costs for a run with four samples are shown. There is a maximum of four samples for the medium TGP on the Illumina Miseq^TM^, therefore only the maximum costs for a run with four samples are given for this type. TGP = targeted gene panel.

**Table S1.** Overview of the NGS test costs with the small TGP.

Step	Activity	Personnel	Time per sample (in min)	Material	Equipment
1	Acceptance coupes of Histology by MD, printing forms, adding details to the MD database.	Secretary	20.00	x	x
2	Histological quality check biopsy, eventually area selection (tumour %)	Technician	5.71	x	€ 1309
		Pathologist	5.00	x	ʺ
3	DNA isolation + Nano drop (quantity)	Technician	14.86	€ 18 per sample	€ 2216
4	Quality check: Quantus (quantity) and QC test (quality)	Technician	13.46	€ 7 per sample	€ 3905
5	TSACP library preparation	Technician	20.36	€ 130 per sample	€ 5434
6	Run NGS, including several checks	Technician	2.14	€ 1,198 per run	€ 30,229 (total costs for the process with the Miseq^TM^ ) / € 196,020 (total costs for the process with the Hiseq^TM^ )
7	Synchronize data outside MiSeq^TM^, check run	Bioinformatician	4.29	x	ʺ
8	Check run, data analysis, set up a conclusion	Technician	30.00	x	€ 51,236
9	Approval conclusion	Molecular Biologist	30.00	x	ʺ
10	Entering data in MD database and patient database	Secretary	15.00	x	x
11	Approval result and lab form	Molecular Biologist	5.00	x	x
		Pathologist	10.00	x	x
		Secretary	10.00	x	x
12	Discussing results with Tumour Sequencing Board (TSB)	Members TSB	1.50	x	x
					€ 2260 (additional costs)

The NGS process for this panel consists of thirteen activities. For each activity, the operating personnel type and the amount of minutes spend per sample, the material costs per sample or run and the equipment costs per year are shown. The material and equipment costs are inclusive VAT and all costs are exclusive overhead. MD = molecular diagnostics, ‘x’ = no material or equipment needed, ‘ ‘’ ‘ = costs are part of the amount mentioned above.

**Table S2.** Overview of the NGS test costs with the medium TGP. The NGS process for this panel consists of fourteen activities.

Step	Activity	Personnel	Time per sample (in minutes)	Material costs	Equipment costs per year
1	Acceptance coupes of histology by MD, printing forms, adding details to the MD database.	Secretary	20.00	x	x
2	Histological quality check biopsy, eventually area selection (tumour %)	Technician	5.71	x	€ 1309
		Pathologist	5.00	x	ʺ
3	Isolation	Technician	12.86	€ 18 per sample	€ 2632
4	Quality check: Nano drop, Qubit (quantity), BioAnalyser (quantity + size)	Technician	8.57	€ 117 per sample	€ 4543
5	Preparation (shearing)	Technician	12.86	ʺ	€ 2991
6	Library preparation	Technician	51.43	ʺ	€ 3216
7	Capture	Technician	19.29	ʺ	€ 30,229 (total costs for the process with the Miseq^TM^) / € 196,020 (total costs for the process with the Hiseq^TM^)
8	Run NGS, including several checks	Technician	2.14	€ 2,427 per run	ʺ
9	Synchronize data outside MiSeq^TM^, check run	Bioinformatician	4.29	x	€ 51,236
10	Check run, data analysis, set up a conclusion	Technician	6.00	x	ʺ
11	Approval conclusion	Molecular Biologist	30.00	x	ʺ
12	Entering data in MD database and patient database	Secretary	15.00	x	x
13	Approval result and lab form	Molecular Biologist	5.00	x	x
		Pathologist	10.00	x	x
		Secretary	10.00	x	x
14	Discussing results with Tumour Sequencing Board (TSB)	Members TSB	3	x	X
					€ 2260 (additional costs)

For each activity, the operating personnel type and the amount of minutes spend per sample, the material costs per sample or run and the equipment costs per year are shown. The material and equipment costs are inclusive VAT and all costs are exclusive overhead. MD = molecular diagnostics, ‘x’ = no material or equipment needed, ‘ ‘’ ‘ = costs are part of the amount mentioned above.

**Table 2.** The molecular diagnostic tests for NSCLC and melanoma patients are shown per hospital type in the old, current and future ­situation.

Time frame	NSCLC Molecular diagnostic tests			Melanoma Molecular diagnostic tests		
**OLD (2012)**						
Peripheral hospitals	Single-gene test (EGFR)	70%	€ 295	Single-gene test (BRAF)	30%	€ 295
Specialised hospitals	Multigene panel < 10 genes (NKI panel) Fragment analysis (HER2/EGFR) FISH (ALK, ROS, RET, or MET)	30%	€ 1,103	HRM (BRAF) Sanger Sequencing (NRAS or KIT)	70%	€ 559
**CURRENT (2015)**						
Peripheral hospitals	Single-gene tests (EGFR + ALK)	70%	€ 559	Single-gene test (BRAF)	30%	€ 295
Specialised hospitals	Small TGP Fragment analysis (HER2/EGFR) Sanger sequencing (EGFR) FISH (ALK, ROS, RET, or MET)	30%	€ 1,504	Small TGP Sanger sequencing (KIT) *in 50% of the patients	70%	€ 753
**FUTURE (2020)**						
Peripheral hospitals	Small TGP	70%	€ 606	Small TGP	30%	€ 606
Specialised hospitals	WGS	30%	€ 1,100	WGS	70%	€ 1100

The targeted gene or panel is shown for each diagnostic test between brackets. NSCLC = non-small-cell lung cancer, FISH = fluorescence *in situ* hybridisation, HRM = high-resolution melt, NGS = next-generation sequencing, TGP = targeted gene panel, WGS = whole-genome sequencing.

**Table S3.** Overview of the molecular diagnostic test costs within the BIA.

Tests	Costs	Cost source
Single-gene test (Sanger sequencing, fragment analysis, sequence analysis)	€ 295	NKI estimation
Single-gene test (HRM)	€ 265	NKI estimation
Single-gene test (FISH)	€ 309	NKI estimation
Multigene panel <10 genes (Sequenom)	€ 500	NKI estimation
TGP small	€ 606	ABC analysis
TGP medium	€ 1,137	ABC analysis
WGS	€ 1,100*	NHGRI [21]

The type of tests, their costs and the source is given. These costs are inclusive VAT and overhead. NKI = Netherlands Cancer Institute, HRM = High Resolution Melt, FISH = Fluorescence In Situ Hybridization, TGP = targeted gene panel, WGS = whole-genome sequencing, NHGRI = National Human Genome Research Institute.

* The NHGRI estimated in October 2015 sequence costs of 1,250 USD per genome. These costs include all production costs, but non-production costs are not included, such as quality assessment and technology development. Per patient four genome sequences, three times the tumour for heterogeneity and once blood as a reference genome, are required to obtain a reliable diagnostic result. In total, this would currently result in WGS test costs of 5000 USD per patient. Since WGS is not yet implemented in the clinic and still under development with major cost reductions over the past years (in 2010 the costs per genome were 29,000 USD), we assume in five years a reduction in costs of 75%. In 2020, this would result in WGS test costs of 1250 USD per patient = ± € 1100.

**Table 3.** Final BIA results, showing the test costs per time frame and showing the budget impact of the current and future situation.

Costs per time frame		NSCLC	Melanoma	Budget impact	Δ NSCLC	Δ Melanoma
**OLD (2012)**	Patient	€ 537	€ 480			
	Population	€ 2,173,812	€ 384,092			
**CURRENT (2015)**	Patient	€ 864	€ 616	**OLD > CURRENT**	€ 327	€ 136
	Population	€ 3,495,055	€ 492,619		€ 1,321,243	€ 108,526
**FUTURE (2020)**	Patient	€ 754	€ 952	**CURRENT > FUTURE**	€ - 110	€ 336
	Population	€ 3,374,582	€ 844,417		€ - 120,473	€ 351,799

The costs are given per patient or for the total stage IV NSCLC and melanoma population within The Netherlands. NSCLC = non-small cell lung cancer.

**Table 4.** Test-related measures, mutation, and treatment characteristics for NSCLC and melanoma patients within the NKI, for whom molecular diagnostic tests were executed in 2 months before NGS implementation and 1.5 months after NGS implementation.

	NSCLC patients			Melanoma patients		
	Before * (*n* = 47)	After * (*n* = 53)	*P* value	Before * (*n* = 36)	After * (*n* = 36)	*P* value
**Test-related measures**						
Average number of tests per patient (min–max)	2.04 (1–4)	3 (1–8)	*P* < 0.05	2.42	1.67	*P* < 0.05
Test costs per patient (min–max)	€ 784 (€ 295–€ 1,623)	€ 1141 (€ 265–€ 2,499)	*P* < 0.05	€ 711 (€ 295–€ 2,851)	€ 698 (€ 500–€ 1,400)	0.331
Test costs per test (min–max)	€ 381 (€ 265–€ 563)	€ 378 (€ 294–€ 606)	*P* < 0.05	€ 294 (€ 265–€ 500)	€ 418 (€ 265–€ 606)	*P* < 0.05
**Mutation characteristics**						
Average number of mutations per patient (min–max) *both actionable and non-actionable	0.71 (0–3)	1.06 (0–4)	0.057	0.75 (0–1)	1.11 (0–3)	*P* < 0.05
Patients containing a mutation for which an EMA approved TT is hypothetically available	7 (16%)	6 (12%)	0.767	14 (39%)	21 (58%)	0.162
Patients containing a mutation for which a TT trial is hypothetically available	11 (24%)	11 (21%)	0.811	0 (0%)	0 (0%)	1.00
**Treatment characteristics**						
Patients started with an EMA approved TT	2 (4%)	3 (5%)	1.00	6 (17%)	3 (8%)	0.479
Patients included in a TT trial	4 (9%)	2 (4%)	0.413	0 (0%)	2 (6%)	0.493
Patients for whom TT was an option, but no start	1 (2%)	5 (9%)	0.211	5 (14%)	1 (3%)	0.199
Patients started in a trial without TT	5 (11%)	2 (4%)	0.246	1 (3%)	0%	1.00
Patients started with a monoclonal antibody	0 (0%)	0 (0%)	1.00	5 (14%)	8 (22%)	0.543
Patients started with a monoclonal antibody trial	0 (0%)	0 (0%)	1.00	2 (6%)	3 (8%)	1.00
Patients started with radiotherapy and/or chemotherapy	15 (32%)	20 (38%)	0.677	8 (22%)	7 (20%)	1.00
Patients starting with an operation	0 (0%)	0 (0%)	1.00	2 (6%)	3 (8%)	1.00
Patients without treatment option	14 (30%)	13 (25%)	0.653	2 (6%)	3 (8%)	1.00
Patients treated in another hospital	6 (13%)	8 (16%)	1.00	5 (14%)	6 (17%)	1.00

For the first four characteristics, the average value and the minimum and maximum are given, for the other characteristics the number of patients and the corresponding percentage is given. NSCLC = non-small-cell lung cancer, EMA = European Medicines Agency, TT = targeted therapy.

**Table 5.** Summary of the BIA case study.

		NSCLC	Melanoma
**Before**	Patient	€ 784	€ 711
	NKI population	€ 268,870	€ 175,622
**After**	Patient	€ 1,141	€ 698
	NKI population	€ 391,368	€ 172,211
**Budget impact**	Patient	€ 357	- € 14
	NKI population	€ 122,498	- € 3,411

The test costs are shown per patient and for the total NKI population in the situation before and after NGS implementation, including the budget impact of the test costs. NKI = Netherlands Cancer Institute.

## References

[1] Vrijenhoek T, Kraaijeveld K, Elferink M (2015). Next-generation sequencing-based genome diagnostics across clinical genetics centers: implementation choices and their effects. Eur J Hum Genet.

[2] Joosten SE, Retèl VP, Coupé VM (2016). Scenario drafting for early technology assessment of next generation sequencing in clinical oncology. BMC Cancer.

[3] Zhao X, Wang A, Walter V (2015). Combined targeted DNA sequencing in non-small cell lung cancer (NSCLC) using UNCseq and NGScopy, and RNA Sequencing Using UNCqeR for the detection of genetic aberrations in NSCLC. PLoS One.

[4] Diamandis M, White NM, Yousef GM (2010). Personalized medicine: marking a new epoch in cancer patient management. Mol Cancer Res.

[5] Hagemann IS, Devarakonda S, Lockwood CM (2015). Clinical next-generation sequencing in patients with non-small cell lung cancer. Cancer.

[6] Gonzalez de castro D, Clarke PA, Al-lazikani B (2013). Personalized cancer medicine: molecular diagnostics, predictive biomarkers, and drug resistance. Clin Pharmacol Ther.

[7] Frampton GM, Fichtenholtz A, Otto GA (2013). Development and validation of a clinical cancer genomic profiling test based on massively parallel DNA sequencing. Nat Biotechnol.

[8] American Cancer Society Non-small cell lung cancer survival rates. http://www.cancer.org/cancer/lungcancer-non-smallcell/detailedguide/non-small-cell-lung-cancer-survival-rates].

[9] American Cancer Society Melanoma skin cancer survival rates. http://www.cancer.org/cancer/skincancer-melanoma/detailedguide/melanoma-skin-cancer-survival-rates].

[10] European Medicines Agency European public assessment reports. http://www.ema.europa.eu/ema/].

[11] Kumarakulasinghe NB, Van zanwijk N, Soo RA (2015). Molecular targeted therapy in the treatment of advanced stage non-small cell lung cancer (NSCLC). Respirology.

[12] Michielin O, Hoeller C (2015). Gaining momentum: New options and opportunities for the treatment of advanced melanoma. Cancer Treat Rev.

[13] Memorial Sloan-Kettering Cancer Center cBioPortal for Cancer Genomics. http://www.cbioportal.org].

[14] Netherlands Comprehensive Cancer Organisation (IKNL) Oncoline treatment guideline NSCLC version 2.1. http://www.oncoline.nl/niet-kleincellig-longcarcinoom].

[15] Netherlands Comprehensive Cancer Organisation (IKNL) Oncoline treatment guideline Melanoma version 2.0. http://www.oncoline.nl/melanoom].

[16] Christensen KD, Dukhovny D, Siebert U (2015). Assessing the costs and cost-effectiveness of genomic sequencing. J Pers Med.

[17] Doble B, John T, Thomas D (2016). Cost-effectiveness of precision medicine in the fourth-line treatment of metastatic lung adenocarcinoma: an early decision analytic model of multiplex targeted sequencing. Lung Cancer.

[18] Li Y, Bare LA, Bender RA (2015). Cost-effectiveness of sequencing 34 cancer-associated genes as an aid for treatment selection in patients with metastatic melanoma. Mol Diagn Ther.

[19] Lievens Y, Van den bogaert W, Kesteloot K (2003). Activity-based costing: a practical model for cost calculation in radiotherapy. Int J Radiat Oncol Biol Phys.

[20] Sullivan SD, Mauskopf JA, Augustovski F (2014). Budget impact analysis-principles of good practice: report of the ISPOR 2012 budget impact analysis good practice II task force. Value Health.

[21] Wetterstrand KA DNA sequencing costs: data from the NHGRI genome sequencing program. www.genome.gov/sequencingcosts].

[22] Bajaj PS, Veenstra DL, Goertz HP (2014). Targeted erlotinib for first-line treatment of advanced non-small cell lung cancer: a budget impact analysis. J Med Econ.

[23] Netherlands Comprehensive Cancer Organisation (IKNL) The Netherlands Cancer Registry. http://www.cijfersoverkanker.nl].

[24] Signaleringscommissie Kanker van KWF Kankerbestrijding (2010). Kwaliteit van kankerzorg in Nederland.

[25] Zorginstituut Nederland Tumor infiltrerende lymfocyten bij patiënten met uitgezaaid melanoom irresectabel stadium IIIc en IV. https://www.zorginstituutnederland.nl/pakket/lopende+dossiers/voorwaardelijke+toelating/tumor-infiltrerende-lymfocyten-bij-patienten-met-uitgezaaid-melanoom-irresectabel-stadium-iiic-en-iv.html].

[26] Signaleringscommissie Kanker van KWF Kankerbestrijding (2011). Kanker in Nederland tot 2020, Trends en prognoses.

[27] Dutch Institute for Clinical Auditing (2015). Dutch melanoma treatment registry: Resultaten van behandeling met nieuwe geneesmiddelen bij gevorderd melanoom. Dutch Institute for Clinical Auditing (DICA) annual report 2014.

[28] Sabatini LM, Mathews C, Ptak D (2016). Genomic sequencing procedure microcosting analysis and health economic cost-impact analysis: a report of the association for molecular pathology. J Mol Diagn.

[29] Kris MG, Johnson BE, Berry LD (2014). Using multiplexed assays of oncogenic drivers in lung cancers to select targeted drugs. JAMA.

[30] Frenkel M (2013). Refusing treatment. Oncologist.

